# Blood pressure response to dynamic exercise testing in adolescent elite athletes, what is normal?

**DOI:** 10.3389/fped.2022.974926

**Published:** 2022-10-21

**Authors:** J.C. Wuestenfeld, F. Baersch, P. Ruedrich, C. Paech, B. Wolfarth

**Affiliations:** ^1^ Department Sports Medicine, Charité – Universitätsmedizin Berlin, Corporate Member of Freie Universität Berlin and Humboldt, Universität zu Berlin, Berlin, Germany; ^2^ Institute for Applied Training Science (IAT), Department of Sports Medicine, Leipzig, Germany; ^3^ Faculty of Sports Science, Institut of Sports Medicine, University Leipzig, Leipzig, Germany; ^4^ Department for Pediatric Cardiology, University of Leipzig –Heart Center Leipzig, Leipzig, Germany

**Keywords:** exercise testing, blood pressure, adolescent athletes, upper limits of blood pressure, sport

## Abstract

**Background:**

In general, only few studies are dedicated to blood pressure behavior under physical stress in children and adolescents. Even less is published about the blood pressure behavior of young high-performance athletes on the ergometer. For this reason, we evaluated the blood pressure behavior under stress compared to non-athletes in a large collective (*n* = 739) of young high-performance athletes (age 10–20 years, mean 15.8 years, male 442, female 297) of different sports. A complete echocardiographic examination was available in all athletes.

**Result:**

Regardless of gender, the young competitive athletes achieved significantly higher maximum blood pressure values than investastigated populations from previous studies. Based on the data obtained, blood pressure percentiles are now defined explicitly for junior athletes across sports as well as age- and gender-dependent, which did not exist in this form of normal values for the special clientele of young competitive athletes. The echocardiographic examinations demonstrated stress-induced cardiac adaptation adaptations in the majority of athletes, which thus correlate with the comparatively higher stress blood pressures compared to non-athletes.

**Conclusion:**

For the first time, blood pressure percentiles for exercise tests on the ergometer for age groups and gender in high performance athletes are defined based on a comparatively large collective of young competitive athletes. Upper limits were determined, in particular for systolic blood pressure under stress, and categorized according to gender and age. Performance diagnosticians and physicians are now enabled to make a more accurate assessment of the corresponding blood pressure regulation of young athletes under exercise conditions.

## Introduction

Arterial hypertension is one of the major treatable cardiovascular risk factors (1). It is associated with elevated mortality and the incidence of heart insufficiency, myocardial infarcts, and apolex (2). Elevated blood pressure in high-performance athletes is one of the most significant risk factors for cardiovascular diseases (3). Exaggerated blood pressure response to exercise testing is commonly regarded as a predictor of developing overt hypertension (4, 5). However, findings in adults are inconsistent (6), and no commonly accepted upper limits indicative of increased risks have been defined so far (7). There are only a few recommendations for tolerable upper blood pressure limits in exercise testing (8, 9). In the Guidelines of the European Society of Cardiology (ESC), it states that an exceeding systolic blood pressure (SB) of 210 mmHg in men and 190 mmHg in women has been termed “exercise hypertension” (7). In the American Heart Association (AHA) guideline for exercise testing (10) a limit of 214 mmHg (based on the 90th percentile calculated from >27,000 treadmill tests (11) is reported beyond which the risk of developing hypertension appears particularly increased (7). Compared to adults, the prevalence of elevated blood pressure in children and adolescents is clearly lower. However, there is a correlation between elevated blood pressure in children and relation to obesity. In contrast to the recommended upper blood pressure limits in adults, the definition of arterial hypertension in children and adults is based on body height and age-dependent limits. These blood pressure limits are determined in healthy children and adolescents. However, such blood pressure limits do not exist for young high-performance athletes, who are exposed to frequent exercise-induced blood pressure increase. There is only limited data available in which the effects of exercise-induced blood pressure elevation have been investigated in children and adolescents. Furthermore, very little is known about the exercise-induced blood pressure response in children. The study by Wanne et al. (12) investigated the blood pressure response under maximal dynamic movement in 497 healthy 9 to 18-year-old on a treadmill in young non- athletes. They described higher systolic values in postpuberty youths than in prepuberty. Szmigielska et al. (13) examined 711 (age 10–18 y) young athletes (training load 7.62 h ± 4.2 h per week). In the maximal testing on the bike ergometer, the SBP was significantly higher in boys than in girls (183.2 ± 27.9 mmHg vs. 170.9 ± 21.4 mmHg, *p* = 0.03). Description of normative response to physical exercise in healthy children and adolescents in terms of percentiles was just recently given by Sasaki et al. and Clark and al. In the study by Clarke and collegues normative percentiles of blood pressure response on a treadmill for healthy children and adolescents were described (14) in contrast to resting blood pressure and individual height which was not considered in the study by Sasaki et al. (15) Although exercise testing in young elite athletes is frequently performed during preparticipation screenings, very little is known about the “normal” magnitude and distribution of exercise-induced blood pressure in this cohort. Therefore, this study aimed to evaluate the magnitude and determinants of blood pressure response to dynamic exercise testing in young elite athletes.

## Materials and methods

### Study population

In the department of sports medicine at the Institute for applied Training Science (IAT) in Leipzig, mainly high-performance athletes, from different sports, are seen mostly for preparticipation screenings. A retrospective analysis of our database from 2010 to 2019 for exercise testing on a bicycle ergometer identified 4,899 athletes. After excluding athletes with an age of >20 years, 2,217 datasets remained. Since the exercise-induced blood pressure regulation was intended to be analyzed in contrast with echocardiographic findings, only exercise tests of athletes were included who had a complete echocardiographic examination within the same year. After data adjustments for doublings, 739 datasets of young athletes remained. Since most of the echocardiographic studies were performed on the day of exercise testing or within a few weeks relevant breaks from sport after injury or illness between exercise testing and echocardiography can ruled out in general. All athletes were highly trained elite athletes of their sports and members of the national team squad according to their age. Non of the tested pupils suffered from any kind of cardiovascular desease nor took any kind of medication that might have an effect on the intrinsic blood pressure reaction

### Anthropometry

Height and weight were measured using a scale with integrated straightedge (Seca scale, Model 701 with telescope measuring stick Model 220, Seca GmbH & Co.Kg. Hamburg, Germany), and body mass index (BMI) was calculated by dividing weight in kilograms through the square of height in meters. Body fat was determined by skinfold thickness using the ten-fold model introduced by Parizkova (16). Fat percentage was then used to partition total body mass into fat mass and fat-free mass. Resting and systolic blood pressure (rSBP) and diastolic blood pressure (rDBP) was measured with a standard sphygmomanometer (Erka, Bad Tölz, Germany) adjusted to the individual's arm circumference on both arms in a sitting position after at least 5 min of resting; the average was calculated for the present study. Exercise systolic blood pressure (eSBP) and diastolic blood pressure (eDBP) was measured with with the similar sphygmomanometer (Erka, Bad Tölz, Germany).

### Exercise testing

Tests were performed on cycle ergometers (Lode sport Excalibur, Lode, Groningen, the Netherlands). The standard protocol for all females started with an initial workload of 50 Watt (W), increment 30 W; stage duration was 3 min. Only petite and lightweight girls started with an initial workload of 25 W. The initial workload for all male athletes was 100 W (increment 30 W, stage duration 3 min). Boys of little height or weight (<50 kg) could also be tested with the girl's protocol. The supervising physician decided the test protocol. All tests were supervised by experienced staff and were performed until subjective exhaustion. All participants were motivated to reach their maximal limits of work capacity by oral motivation. Sine all participants were experienced athletes, all participants were familiar with voluntary maximal exhaustion. Heart rates and BP were measured at the end of each stage, including maximal SBP and DBP (mSBP; mDBP). Heart rates were obtained automatically from 12-channel ECG (cardio 300, Custo med GmbH, Ottobrunn, Germany) but were verified and corrected (if necessary) manually afterward. BP was measured manually using standard sphygmomanometers adjusted to the individual's arm circumference.

### Echocardiography

Experienced echocardiographers performed two-dimensional echocardiography according to guidelines of the german society of cardiology valid for the study period (Compare: Hagendorff et al.; Manual zur Indikation und Durchführung der Echokardiographie). From 2010 to 2014, Philips IE 33 system (Philips Healthcare, Hamburg, Germany) and after 2014 a Philips Epiq, both with a 3.5 MHz transducer. Left atrial diameters (anteroposterior) were assessed from either B-mode or M-mode parasternal long-axis views. LV end-diastolic diameter and septal wall thickness were measured in the parasternal long-axis at the level of the LV minor axis, approximately at the mitral valve leaflet tips. The absolute and relative mass of the heart was calculated using the formula established by (17) Pulsed Doppler profiles at the distal margins of the mitral valve leaflets, deceleration time of the E wave and, tissue-Doppler-derived mitral annular velocities (average of septal and lateral E′) were assessed as indices of diastolic function. Movement in the M-mode (TAPSE and MAPSE) further assessed the function of the right and the left ventricle. For the relevant echocardiographic of this study z-scores were calculated. During the time of the study interval overall 3 physicians where involved in echocardiographic data gathering. one of the examiners was a specialist for sports medicine with more than 20 years experience in echocardiography, one was cardiologist and sports cardiologist with the highest certified level of training in transthoracal echokardiograhy and one was resident physician in training for internal medicine and sports medicine who was trained and observerd in echocardiography by the cardiologist. Complete echocardiographic data were available for all athletes of the presented study.

### Sporting disciplines and training intensity

The classification of sports by Schnabel and Thieß (18) subdivides disciplines in technical-acrobatic sports, double-fight, endurance, sprint power/ strength and game sports. Based on this, all athletes were classified in one of these clusters to compare sport related differences in exercise related BP reaction. Furthermore, all athletes were clustered for their individual training intensity (by training hours per week) 4 clusters of training intensity were defined: 1–5, 6–10, 11–15 and 16–20 h/week. Given that not only the training intensity but also the age has an impact on the regulation of the BP during exercise, the component of age was considered separately. Athletes aged 10–12 years, 13–15 years, 16–18 years, and 18–20 years were clustered for age-dependent examinations.

### Statistical analysis

The data obtained from the stress tests and echocardiographic examinations were processed in Microsoft Office Excel 2016 for Windows from Microsoft Corporation (Redmond, USA) and statistically calculated using IBM SPSS Statistics 25.

Except for age, all data fulfilled criteria for normal statistical distribution as verified by previous authors (19) thus consistent descriptive presentation as meanSD was chosen for all variables including age. Mean comparisons were calculated with a single factorial variance analysis. Due to the literatur (20), the robust Welch test was also tested for corresponding significance. In significant differences, a posthoc test and Bonferroni correction were calculated. The significance level was = 0.05. According to Cohen (1988), the effect sizes were interpreted as follows:.01 small effect,.06 medium effect, and.14 large effect. Correlation effects are described by the correlation coefficient |*r*|. According to Cohen Guidelines (1988) data was interpreted as: |*r*| = 0.10 a weak |*r*| = 0.30 a moderate |*r*| = 0.50 describe a strong correlation. Associations of variables were analyzed using Pearson's correlation coefficients. Normative ranges were defined as the central 95% of all observations, and lower and upper limits were estimated assuming normality as means 2-fold SD. Additionally, 95% confidence intervals for limits of normative ranges are presented in squared brackets [e.g., (1234)].

## Results

### Study population

A total of *n* = 739 athletes (male = 442, female = 297) with a median age of 15.8 years (±2,3 years) were analyzed. There was no significant difference in age and duration of training years between male and female athletes. The demographic and clinical characteristics of this population are summarized in Table 1. Male athletes were significantly heavier and taller than their female counterparts, had less body fat, a higher fat-free mass (FFM), and a higher resting blood pressure rSBP and rDBP (Compare Table 1)

**Table 1:** Baseline anthropometric and clinical characteristics of study population.

	All	Male	Female	*p*-value
Number of participants	739	442 (59.8%)	297 (40.2%)	
Age (years)	15.8 ± 2.3	15.9 ± 2.8	15.6 ± 2.4	*p* = 0.149
Years of training	6.7 ± 2.8	6.7 ± 2.8	6.7 ± 2.6	*p* = 0.834
Hrs. of training/week)	14 ± 6.2	13.8 ± 6.3	14.4 ± 6	*p* = 0.194
BMI (kg/m^2^)	21.2 ± 3.2	21.3 ± 3.2	21.1 ± 3.1	*p* = 0.370
Height (cm)	175 ± 11.3	179 ± 11.1	168 ± 7.6	*p* < 0.001
Weight (kg)	65.5 ± 14.8	69.3 ± 15.5	59.9 ± 11.5	*p* < 0.001
Fat mass (kg)	10.1 ± 4.6	9.2 ± 4.1	11.3 ± 4.9	*p* < 0.001
Body fat %	15.3 ± 5.0	13.2 ± 4.0	18.4 ± 4.7	*p* < 0.001
Lean mass (kg)	55.5 ± 12.5	60.1 ± 13	48.7 ± 7.9	*p* < 0.001
Resting SBP (mmHg)	121 ± 12	124 ± 13	117 ± 10	*p* * < 0.001*
Resting DBP (mmHg)	78 ± 8	80 ± 8	76 ± 8	*p* * < 0.001*

### Echocardiographic findings at baseline

An overview of the echocardiographic data is shown in Table 2 below. Z-scores of the relevant echocardiographic results are given in Table 3. The absolute cardiac size in male athletes was significantly higher (815.4 ± 188.2 g) than in female athletes (637.7 ± 115.4 g). In relation to the body weight, the male heart volume was also higher 11.85 ± 1.54 g/kg body weight than that of the female 10.79 ± 1.56 g/ kg body weight. There were no significant differences in both the medial and lateral E wave and the right ventricular function in the form of TAPSE (tricuspid annular plane systolic excursion). The diameter values of the left ventricular posterior wall during the diastole is within the normal range for both female and male post-growth athletes. The male subjects have significantly higher wall thicknesses of 0.99 ± 0.15 cm than the female subjects with 0.88 ± 0.13 cm (*p* < 0.001). The diameter of the left atrium 3.42 ± 0.43 (♂), bzw. 3.2 ± 0.41 cm (♀) was within the standard range. The values of male athletes were significantly higher than those of female athletes [0.221; 95% CI (0.158. 0.283)]. The differences between the sexes in the left ventricular diameter's values during the diastole (LVEDD) and the interventricular septum thickness were significantly different, too (both with *p*.001). All subjects were in the standard range with their respective values. All relevant echocardiographid data are presented in relation to athletes age and sex in Figures 4–11.

**Table 2:** Echocardiographic characteristics of study population.

variable	all	♂	♀	*p*-value
e′-wave lateral (cm/s)	5.16 ± 1.08	5.12 ± 1.07	5.22 ± 1.11	*p* = 0.210
e′-wave medial (cm/s)	7.46 ± 1.50	7.40 ± 1.48	7.55 ± 1.53	*p* = 0.191
Heart volume absolute (g)	743.9 ± 184.7	815.4 ± 188.2	637.7 ± 115.4	*p* < 0.001
Relative heart volume (g/kg)	11.42 ± 1.64	11.85 ± 1.54	10.79 ± 1.56	*p* < 0.001
Left ventr. posterior wall (cm)	0.95 ± 0.15	0.99 ± 0.15	0.88 ± 0.13	*p* < 0.001
TAPSE (cm)	2.43 ± 0.38	2.44 ± 0.38	2.40 ± 0.38	*p* = 0.228
Endsystolic left atrial diameter (cm)	3.33 ± 0.44	3.42 ± 0.43	3.20 ± 0.41	*p* < 0.001
Left ventr. enddiastolic diameter (cm)	4.90 ± 0.47	5.05 ± 0.46	4.68 ± 0.39	*p* < 0.001
Enddiastolic septal wall (cm)	0.90 ± 0.13	0.94 ± 0.13	0.84 ± 0.11	*p* < 0.001

**Table 3:** Z-scores of echocardiographic results.

Age group	Sex	Mean value			
		IVSd-Z	IVSs-Z	LVIDd-Z	LVIDs-Z
10–12	M	0.849	1.109	0.077	0.035
10–12	W	0.689	0.757	0.102	0.113
13–15	M	0.757	1.261	0.345	0.266
13–15	W	0.469	0.840	0.130	0.079
16–17	M	0.766	1.454	0.070	0.169
16–17	W	0.481	0.895	−0.173	−0.094
18–20	M	1.166	1.842	−0.111	0.222
18–20	W	0.938	0.840	−0.270	0.139

### Exercise-induced blood pressure response

Table 4 summarizes the findings related to exercise testing. SBP increased significantly (*p* < 0.001 for both sexes). whereas DBP remained almost unchanged in femal and male athletes. Male athletes absolved more exercise stages, started with a higher workload, attained higher peak workloads, and showed a more pronounced SBP response than female athletes

**Table 4:** Findings in exercise testing.

	All	♂	♀	*p*-value
Initial workload (watt)	75.5 ± 10.6	92 ± 19	52 ± 9	*p* < 0.001
Max workload (watt)	231 ± 68	258 ± 67	190 ± 45	*p < 0.001*
Max workload relative (watt/kg)	3.5 ± 0.8	3.8 ± 0.7	3.2 ± 0.7	*p < 0.001*
Max lactat (mmol/L)	8.7 ± 2.4	8.8 ± 2.4	8.3 ± 2.5	*p* = 0.015
Max SBP (mmHg)	187 ± 23	193 ± 23	177 ± 19	*p* < 0.001
Max DBP (mmHg)	80 ± 9	81 ± 8	79 ± 9	*p < 0.001*
Max. heart rate (x/min)	189 ± 11	189 ± 11	189 ± 11	*p* = 0.622

### Influence of age on the maximum of exercise-induced blood pressure

**Figure 1:**
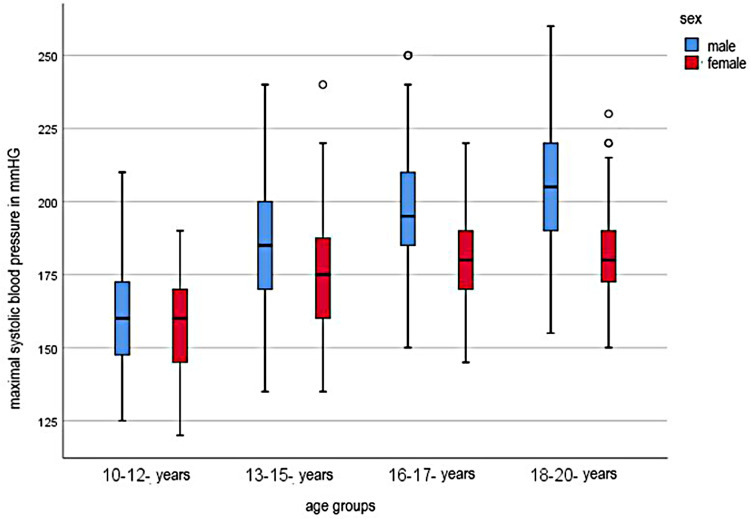
Systolic maximal blood pressure for age groups.

In the cohort of 10–12 year old junior athletes, the SBP at maximal effort was 159 mmHg (±18 mmHg). In the progression of the age of the athletes, the SBP increased continuously. 13–15 year old athletes had an average SBP of 182 ± 20 mmHg, 16–17 year old athletes 192 ± 20 mmHg, and 18–20 year old athletes 196 ± 22 mmHg. There was a significant difference between male and female athletes in which male athletes gained higher values. Within the different age cohorts, the SBP was also significantly different between sexes. (see Figure 1)

### Influence of training frequency

**Figure 2:**
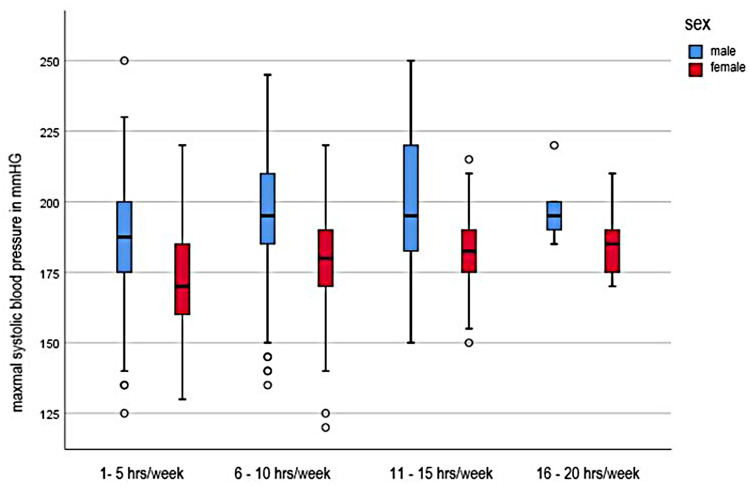
Systolic maximal blood pressure in dependence of training intensity.

The influence of the training frequency on the maxSBP is shown in Figure 2. The statistical calculation showed that there were significant differences between the frequency of the training and the blood pressure development of the subjects (*F* = 11.823, *p* < 0.001). The post-hoc comparison of the categories of these significant differences between the lowest training frequency of 1–5 h/week and 6–10 h/week (−11.161; 95% CI [−16.43; −5.89] and between a training effort of 1–5 h and 11–15 h per week (−14.754; 95% CI [−23.07; −6.44]. The maximum systolic blood pressure of children and adolescents is initially linear. From a training range of 6–10 h, the values flatten and the blood pressure increases only minimally in both sexes. The maximum values are reached with a training range of 16–20 h.

### Influence of sporting type

To investigate the influence of the sporting type, the athletes were divided into sporting type groups according to Schnabel (1993) in technical-acrobatic sports, double-fight, endurance, sprint power/strength and game sports. The maximum systolic blood pressure values differed significantly in terms of the respective sports (*F* (4;732) = 4.850, *p* = 0.001). The highest mSBP values were observed in the sprint/ strength athletes with an average value of 190 (±23) mmHg. The lowest values were recorded by athletes from technical-acrobatic sports with an average mSBP value of 178 (±21) mmHg. Significant differences were found in the maximum systolic blood pressure values between the technical-acrobatic and endurance sports (−9.657; 95%-CI [−17.12; 1.04] as well as the technical-acrobatic and rapid-power or strength athletes (−12.653, 95%-CI [−21.147; −3.83]. In both cases, the technical-acrobatic athletes had lower mSBD values. Compare Table 5.

**Table 5:** Influence of sporting type.

Type of sport	6	mSBD (MW ± SD)
Technical-acrobatic sports	97	178 ± 21
Double-fight	100	178 ± 21
Endurance	316	187 ± 23
Sprint power/ strength	113	190 ± 23
Game sports	112	189 ± 23

### Association of the mSBP with cardiac dimensions

The Pearson correlation coefficient shows a moderate positive correlation between the relative heart volume (g/kg BM) and the mSBD (*r* = 0.171). The thicker the interventricular septum during the junior athletes' diastole, the higher the systolic rest values were (*r* = 0.323). The effect can be interpreted as moderate. If one combines above two echo parameter with the maximum systolic blood pressure value, the effect (*r* = 0.490) strengthens and indicates that the higher the echocardiographic diameters, the higher the mSBD values during the load.

**Figure 3:**
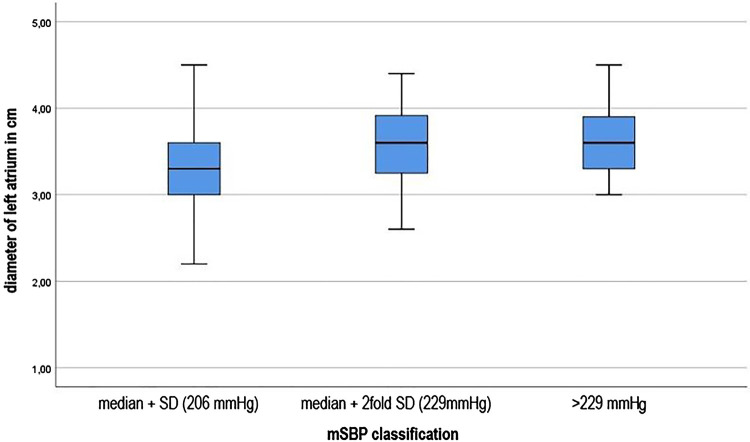
Left atrial dimension in dependence of increasing deviation from the mean (SBP).

Finally, it was investigated whether those young athletes who had significantly higher systolic blood pressure levels during the stress test also showed corresponding abnormalities in cardiac ultrasound. (see Figure 3) Significant differences of the mean SBP were found in the statistical calculation of the left ventricular diameter during the diastole, *F* (3; 738) = 27.552, *p*.001 effect strength high (*η*^2 ^= 0.101) (Cohen, 1988). It is noticeable that with increasing deviation from the mean, the diameters in the left ventricle also increase during the diastole. Those young athletes who had a maximum systolic blood pressure value >229 mmHg also showed the highest end-diastolic diameter. The differences were highly significant (*F* (3.738) = 28.134, *p*.001) with also high effect strength (*η*^2 ^= 0.103). Both between the single and the double SD (−0.0983; 95%-CI [−0.136; −0.061]) and between the simple and the more than double SD, i.e., a systolic blood pressure development of more than 229 mmHg (−0.1376; 95%-CI [−0.193; −0.082]), the calculations showed highly significant results. Equally significant were the calculations of the left ventricular posterior wall thickness (*p*.001). Similar to the above results, significant differences between the single and the double SD (−0.1099; 95% CI [−0.152; −0.068]) and between the simple SD and the classification >229 mmHg (−0.1374, 95% CI [−0.2; −0.075]) were found. Concerning right ventricular function in the form of TAPSE, the groups do not differ significantly (*p* = 0.089). The left atrium diameter showed significant differences between the mSBD classifications, *F* (3. 727) = 12.308, *p*.001. The post-hoc testing significant differences between the group of athletes with single SD and double SD (−0.236; 95%-CI [−0.365; −0.108]) and between single SD and the classification group “blood pressure >229 mg” (−0.288; 95%-CI [−0.478; −0.096]).

**Figure 4:**
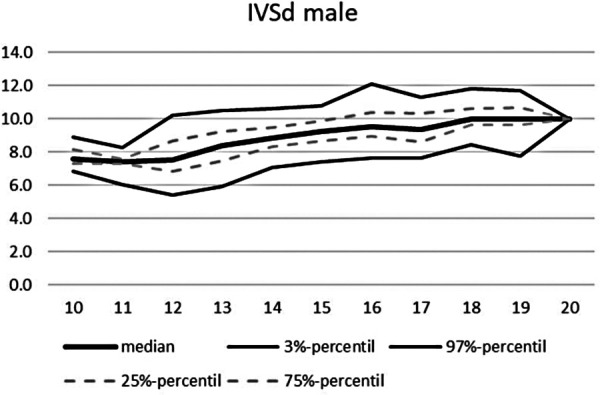
Echocardiographic values in dependence of age, IVSd male.

**Figure 5:**
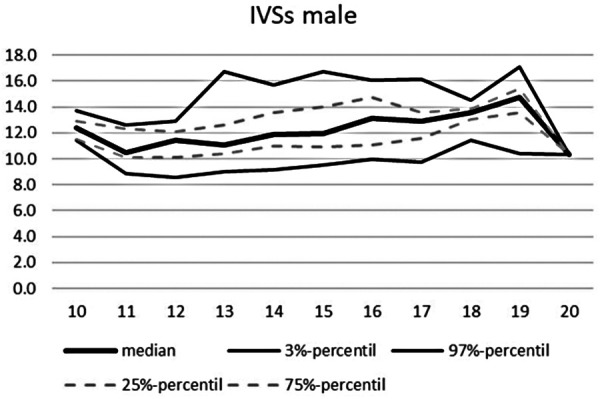
Echocardiographic values in dependence of age, IVSs male.

**Figure 6:**
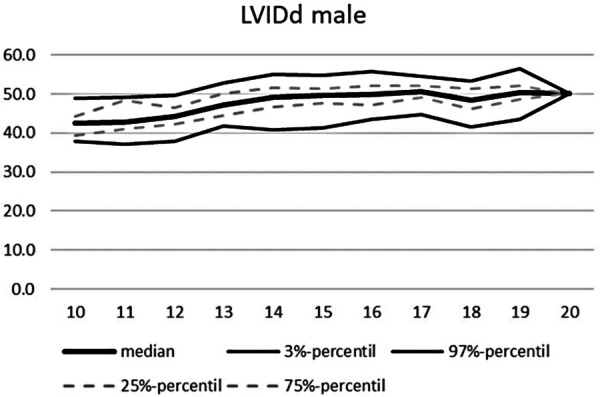
Echocardiographic values in dependence of age, LVIDd male.

**Figure 7:**
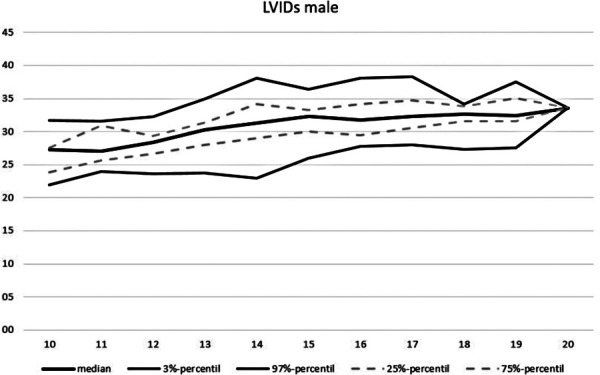
Echocardiographic values in dependence of age, LVIDs male.

**Figure 8:**
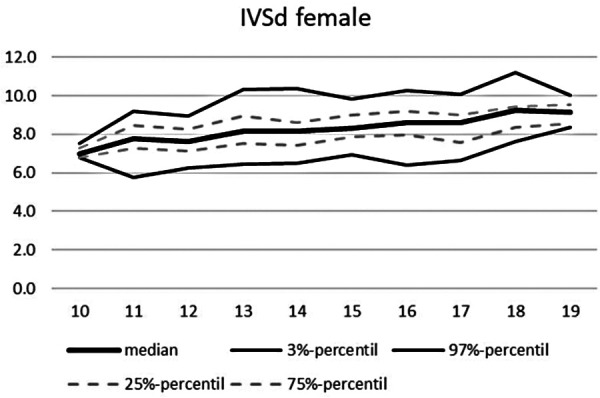
Echocardiographic values in dependence of age, IVSd female.

**Figure 9:**
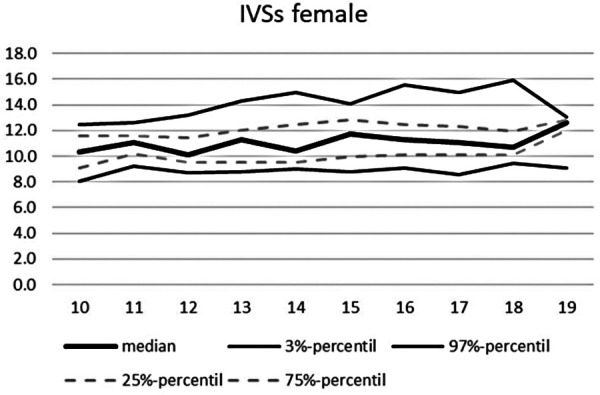
Echocardiographic values in dependence of age, IVSs female.

**Figure 10:**
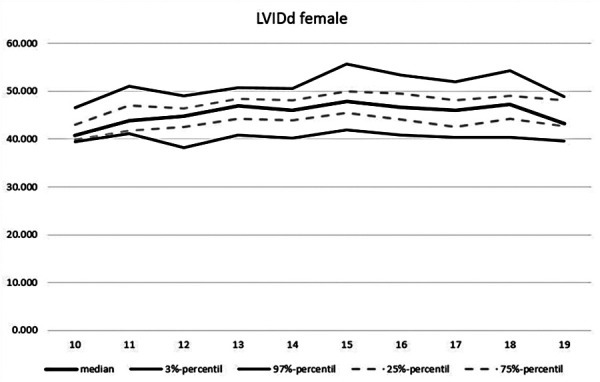
Echocardiographic values in dependence of age, LVIDd female.

**Figure 11:**
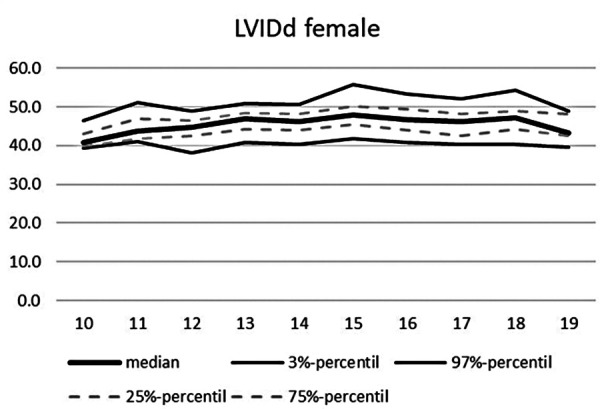
Echocardiographic values in dependence of age, LVIDd females.

### Blood pressure percentiles

Of the 739 studied young athletes, percentiles of blood pressure levels were calculated. These were initially calculated jointly for both sexes and all age groups. Within the 95th percentile are blood pressure values diastolic of 95 mmHg and systolic of 230 mmHg. Across all age groups and separated by gender, 95% of young male athletes aged 10–20 years have a blood pressure below 230/95 mmHg. For female athletes, the 95th percentile is 206/90 mmHg.

In addition to distinguishing blood pressure limits by sex, age-dependent distinctions are of particular interest. Thus, all subjects were additionally assigned to a corresponding age group. For each age group, blood pressure limits have now been determined by calculating the percentiles, which can quickly provide information about possible exercise-induced hypertonia (see Tables 6–8).

**Table 6:** Age dependent blood pressure percentiles of maximal systolic blood pressure in mmHg for male and female junior athletes.

Percentiles	Age			
	10–12-years *n* = 91	13–15-years *n* = 342	16–17-years *n* = 230	18–20-years *n* = 90
75. percentile	173 mmHg	195 mmHg	200 mmHg	215 mmHg
90. percentile	180 mmHg	210 mmHg	220 mmHg	227 mmHg
95. percentile	190 mmHg	217 mmHg	235 mmHg	230 mmHg
99. percentile	—	236 mmHg	248 mmHg	247 mmHg

**Table 7:** Age dependent blood pressure percentiles of male junior athletes.

Percentiles	Age			
	10–12-year old	13–15-year old	16–17-year old	18–20-year old
75. percentile	175 mmHg	200 mmHg	210 mmHg	220 mmHg
90. percentile	181 mmHg	210 mmHg	230 mmHg	230 mmHg
95. percentile	200 mmHg	220 mmHg	240 mmHg	240 mmHg
99. percentile	—	237 mmHg	250 mmHg	258 mmHg

**Table 8:** Age dependent blood pressure percentiles of female younior athletes.

Percentiles	Age			
	10–12-year old	13–15-year old	16–17-year old	18–20-year old
75. percentile	171 mmHg	190 mmHg	190 mmHg	190 mmHg
90. percentile	183 mmHg	200 mmHg	200 mmHg	200 mmHg
95. percentile	190 mmHg	208 mmHg	200 mmHg	217 mmHg
99. percentile	—	238 mmHg	—	—

## Discussion

This work aimed to investigate the blood pressure behavior of young athletes on the bicycle ergometer and to establish blood pressure limits that apply to young competitive athletes. In contrast to many exercise testing in international studies ergometry was conducted on the bicycle ergometer. Since the reason for exercise testing was focused on preparticipation screening in sports the quality of the exercise ECG to detect any cardiac pathology is essential. In terms of torso movement related ECG artefacts the ECG quality is much higher than during an exercise ECG testing on a treadmill.

According to the guidelines of the European Society of Hypertension (ESH), the resting blood pressure values of the athletes are within the normal range (21). In this study, male athletes had slightly higher resting blood pressure levels on average than female athletes. The studies of Pressler et al. (7) and Al-Sendi et al. (22) confirmed these gender differences in resting blood pressure levels. These gender differences are thought to be hormonal (23). The blood pressure progression of our subjects until exhaustion is similar to that described in previous studies (7, 24). The systolic blood pressure increases linearly with the load in all subjects. Diastolic blood pressure, on the other hand, flattened by a maximum of 22 mmHg from start to the last stage (25). However, diastolic blood pressure under maximum stress is often measured incorrectly too low using the Riva Rocci method (26)

The sex, the age, the proportion of fat-free mass and the amount of training exercise influence the development of the maximum systolic blood pressure. Male athletes had higher mSBD levels and an equally higher fat-free mass than their female counterparts did. It is believed that the higher weight associated with increased muscle mass allows for higher performance on the ergometer. The extent of the increase in systolic blood pressure is also related to the mass of muscles involved (25). However, if one looks at the relative workload per kilogram, the difference between the sexes is virtually non-existent. As the relative workload increases, so does the maximum blood pressure.

That the age component has a decisive influence on the development of maximum systolic blood pressure under stress (27), was confirmed in this study. In the Kiel EX. PRESS study (27) reference values for systolic blood pressure limits derived from bicycle ergometry tests were described. In addition, our study found an influence of age on the development of maximum systolic blood pressure values. The oldest study group with an age of 18–20 years had the highest maximum blood pressure values (196 ± 22 mmHg), the youngest subjects (10–12 years) the lowest. Since this data is collected on high-performance athletes who are (almost) daily exposed to their sports specific training, our values are significantly higher than previously described (27)

The training frequency parameter had a significant influence on the maximum systolic blood pressure development of young athletes. It confirmed the results of the meta-analysis of Berge et al. (2015). The research team around Berge classified the training amount into two categories: >10 h per week and 10 h of training effort per week. We were able to confirm the fact that those athletes who exercised more frequently (>10 h/week) had higher blood pressure development. With a four-part classification of the training frequency (1–5 h, 6–10 h, 11–15 h, 16–20 h), a linear relationship between training frequency and mSBD development could be revealed across genders. However, what is striking about both sexes is that the mSBD forms a plateau from a training range of 6–10 h and then only increases minimally with increasing training scope. The maximum values are reached for both sexes with a training range of 16–20 h. These results suggest that from a training volume of 6 h per week, sports-related adjustment reactions of blood pressure occur.

Influence of the sports type can be found as well. The technical-acrobatic sports results were significantly different compared to endurance athletes and sprint/strength and strength sports. In both cases, the technical-acrobatic athletes had a lower maximum systolic blood pressure level. This result differs from other study results (3, 28). We assume that a very different composition of sports can explain these divergent results. In addition, in some sports, unattained maximal cardiovascular stress due to premature muscular fatigue must be assumed. Premature test cancellation meant that maximum blood pressure might not have been reached.

When looking at the subjects with the highest systolic blood pressure values in the exhaustion, significant abnormalities could be identified in single athletes. A canoeist and a cross-country skier had a maximum value of 260 and 250 mmHg, by far the highest systolic values. Both athletes also performed correspondingly high but not the highest relative workloads. However, it is also noticeable that in both athletes, the systolic resting blood pressure was above the age-appropriate limit. Excitement, but also cardiovascular regulation disorders, can be the cause of this.

On the contrary, by looking at those athletes who stand out with a particularly good relative performance, it becomes clear that in these subjects, the maximum systolic blood pressure only assumes values of 200 mmHg. All these athletes are even in their 90th percentile of upper systolic blood pressure levels. As a result, no direct linear relationship can be determined between an excessive blood pressure reaction and a higher relative performance on the bicycle ergometer.

Overall, the majority of the athletes studied (*n* = 610, 82,5%) are in the range of the calculated mean value plus the simple standard deviation in terms of the maximum systolic blood pressure reaction (<206 mmHg). According to the calculated percentiles, this corresponds to the 90th and 95th percentiles in all age ranges. Ninety-one subjects had mSBD values above the mean up to twice the standard deviation (up to 229 mmHg).

In 38 of the 739 athletes studied, the systolic maximum values during the maximal workload were even higher than a deviation of twice the SD of the mean (>229 mmHg). Thirteen of the 38 athletes with excessive blood pressure reaction above systolic values of 229 mmHg correspondingly had norm-deviating systolic rest values of >140 mmHg. Whether these deviations are excitement-related increases, or already an indication of aterial hypertension, is to be discussed.

Those young athletes who had a maximum systolic blood pressure value >229 mmHg showed significant different (higher) values for inner left ventricular dimension in the systolic and diastolic and the left ventricular posterior wall thickness. Therefore these athletes might need to be monitored more closely during their career, if both cardiac function and dimensions and blood pressure regulation show any signs of pathologic development.

Due to the increase in blood pressure and the associated increase in intramyocardial pressure, there may be increased wall stress on the heart during a training career [see, among others (29)]. Echocardiography is an important examination methodology for assessing and demarcating a pathological adaptation from a physiological (sport-related) adaptation. Overall, the results from the statistical calculations show a few echocardiographic abnormalities, although the vast majority of athletes are within the norm values for excluding pathological cardiac changes.

### Limitations

However, since this work is only a cross-sectional analysis, the long-term effects of stress-induced hypertension are neglected. Mainly the long-standing competitive sport with numerous intensive training sessions and competitions provokes functional and structural changes to the musculoskeletal system and the cardiovascular system (30). To rule out pathological changes due to intensive training, further, regular examinations must take place for each athlete during his/her career. Only in this way, can the influence of a long-term high training workload reveal individual cardiovascular risks and provide information about how the body deals with high blood pressure provoked by training in the long term.

The presented blood pressure percentiles for children and adolescents are related to sex and age to simplify the graphic representation. The aspect of body hight, which is known to effect the age dependent resting blood pressure was not taken into account and might lead to misinterpreting the exercise blood pressure children and adoslescents.

Another limitation of this work is that a pre-test activity, the nutritional status of the athletes, but also temporary emotional and psychological stress, such as in school or family, were not taken into account. However, all these factors can influence athletic performance and resulting blood pressure. The extent to which they have an impact remains debatable.

To define cluster of paediatric subjects by age may not be appropriate when investigating the influence of physical maturity on parameters. Unfortunatly no data of puberty was gathered during the regular clinical assessment. Therefore the influence of pre- or late puberty on exercise related blood pressure regulation cannot be assessed in this study.

## Conclusion

In this study, the blood pressure behavior of young athletes aged 10–20 years was investigated and evaluated retrospectively. Overall, the young athletes show standard-compliant systolic and diastolic rest blood pressure values in the results of the examination. Only the level of maximum blood pressure values differs between athletes and non-athletes.

For the first time, blood exercise related pressure percentiles were defined for junior athletes across sports. Competitive athletes in the junior field achieve higher blood pressure values due to physiological training adaptations and tolerate overall higher maximum blood pressure values than subjects who do not exercise.

However, children and adolescents who are above these limits during exercise exposure may be at higher risk of cardiovascular disease.

Overall, a large proportion of the athletes studied already show cardiac adaptation reactions that have a positive effect on performance. However, those subjects who had excessive BD reactions showed significantly higher chamber septum thicknesses during the diastole and higher left ventricular posterior wall thicknesses compared to their colleagues. Although these values are still within the range of the athlete-specific norm values, a tendency related to blood pressure is visible. Regular medical examinations should further be carried out to ensure the long-term health of all athletes.

In general, there are only a few studies devoted to blood pressure behavior in childhood and adolescence. There is a need for research, especially to the question of what causes stress-induced hypertension in the long term. For this purpose, further longitudinal studies should be done examining children and adolescents from junior performance sports several times during their sporting careers for abnormalities.

## Data Availability

The raw data supporting the conclusions of this article will be made available by the authors, without undue reservation.
